# TRPC Channels in the SOCE Scenario

**DOI:** 10.3390/cells9010126

**Published:** 2020-01-05

**Authors:** Jose J. Lopez, Isaac Jardin, Jose Sanchez-Collado, Ginés M. Salido, Tarik Smani, Juan A. Rosado

**Affiliations:** 1 Department of Physiology (Cell Physiology Research Group), Institute of Molecular Pathology Biomarkers, University of Extremadura, 10003 Caceres, Spain; josesc@unex.es (J.S.-C.); gsalido@unex.es (G.M.S.); jarosado@unex.es (J.A.R.); 2 Department of Medical Physiology and Biophysics, Institute of Biomedicine of Sevilla, 41013 Sevilla, Spain; tasmani@us.es

**Keywords:** TRPC1, STIM1, Orai1, calcium influx, store-operated Ca^2+^ entry (SOCE)

## Abstract

Transient receptor potential (TRP) proteins form non-selective Ca^2+^ permeable channels that contribute to the modulation of a number of physiological functions in a variety of cell types. Since the identification of TRP proteins in *Drosophila*, it is well known that these channels are activated by stimuli that induce PIP_2_ hydrolysis. The canonical TRP (TRPC) channels have long been suggested to be constituents of the store-operated Ca^2+^ (SOC) channels; however, none of the TRPC channels generate Ca^2+^ currents that resemble *I*_CRAC_. STIM1 and Orai1 have been identified as the components of the Ca^2+^ release-activated Ca^2+^ (CRAC) channels and there is a body of evidence supporting that STIM1 is able to gate Orai1 and TRPC1 in order to mediate non-selective cation currents named *I*_SOC_. STIM1 has been found to interact to and activate Orai1 and TRPC1 by different mechanisms and the involvement of TRPC1 in store-operated Ca^2+^ entry requires both STIM1 and Orai1. In addition to the participation of TRPC1 in the *I*_SOC_ currents, TRPC1 and other TRPC proteins might play a relevant role modulating Orai1 channel function. This review summarizes the functional role of TRPC channels in the STIM1–Orai1 scenario.

## 1. Introduction

**Figure 1:**
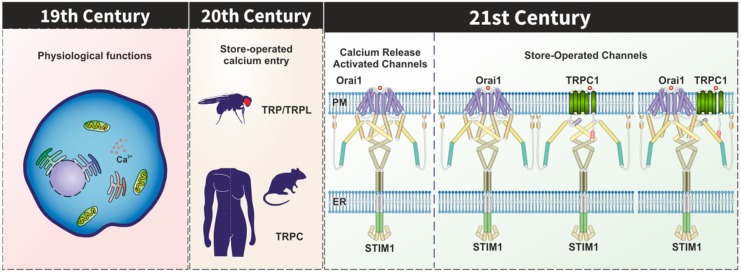
Milestones in the characterization of Ca^2+^ entry. In the early 1880s, Ringer revealed the functional role of Ca^2+^ entry in heart contraction. About a century later, store-operated Ca^2+^ entry (SOCE) was discovered and, by that time, transient receptor potential (TRP) channels were identified, first in *Drosophila* and then in mammals. In 2005 and 2006 STIM1 and Orai1, the key components of the Ca^2+^ release-activated Ca^2+^ (CRAC) channels, were identified, and canonical TRP (TRPC) channels were found to participate in a non-selective store-operated current together with STIM1 and Orai1. The model represents two alternatives for the involvement of TRPC in the store-operated channels.

The relevance of Ca^2+^ influx in cellular physiology was revealed by Ringer in the early 1880s [1] and was almost a century later when store-operated Ca^2+^ entry (SOCE), also known as capacitative Ca^2+^ entry, was identified [2] (Figure 1). SOCE is a singular mechanism for Ca^2+^ influx as it is activated by discharge of the intracellular agonist-sensitive Ca^2+^ stores unlike other Ca^2+^ entry pathways activated by physical changes of the plasma membrane (PM) or direct chemical stimulation of the channels. A number of store-operated currents with different biophysical properties have been described; among them, the first identified and best characterized one is the highly Ca^2+^ selective Ca^2+^ release-activated Ca^2+^ current (*I*_CRAC_). *I*_CRAC_ is a non-voltage activated, inwardly rectifying, current initially described in mast cells upon depletion of the intracellular Ca^2+^ pools by means of stimulation with inositol 1,4,5-trisphosphate (IP_3_), ionomycin, or excess of EGTA [3]. As mentioned before, *I*_CRAC_ is not the only store-operated current and a variety of store-operated currents grouped under the term *I*_SOC_ have been reported in different cell types, which differ from *I*_CRAC_ in several biophysical features (see Table 1), including that *I*_SOC_ are not selective for Ca^2+^ and exhibit greater conductance than *I*_CRAC_ (for a review, see [4]). Since the identification of SOCE, two main issues attracted considerable attention and interest: (1) the molecular basis of the communication between the intracellular Ca^2+^ stores and the channels in the PM and (2) the nature of the store-operated channels.

Back to 1969, Cosens et al. identified a spontaneous *Drosophila* mutant with altered electroretinogram [5] that was attributed to a mutation of the so called transient receptor potential (TRP) channel that resulted in transient, rather than sustained, light-dependent depolarization of the photoreceptors upon Na^+^ and Ca^2+^ entry [6]. *Drosophila* TRP and its homologue TRPL were characterized as Ca^2+^ permeable channels activated downstream of phospholipase C [7]. In 1995, two separate groups identified the first human homolog of the *Drosophila* TRP channel, TRPC1 [8,9]. After the characterization of TRPC1, a number of homologs were identified in mammalian cells and grouped into six subfamilies: TRPC (canonical) comprising seven members (TRPC1-TRPC7), TRPV (vanilloid) including subtypes TRPV1 to TRPV6, TRPM (melastatin), which comprises eight members (TRPM1-TRPM8), TRPA (ankyrin) consisting of only one member TRPA1 and TRPP (polycystin) as well as TRPML (mucolipin) comprising three members each (revised in [10,11]).

The basic structure of TRP channels consists of six transmembrane helical domains (TM1 through TM6) with a loop between TM5 and TM6 forming the channel pore and *N*- and *C*-terminal regions located in the cytosol. TRP channels are thought to tetramerize to form a 24-helix functional protein complex. Mammalian TRP channels exhibit different functional domains, including a variable number of *N*-terminal ankyrin repeats present in TRPC, TRPV and TRPA that is involved in protein-protein interaction (revised in [10,12,13]). Remarkably, three members of the TRPM subfamily contain a catalytic kinase domain in the *C*-terminal region and TRPC and TRPM channels exhibit a conserved TRP domain adjacent to TM6, containing a highly conserved sequence named TRP box, involved in signal transduction coupling and channel gating [14]. In addition, a number of mammalian TRP members contain *N*- and/or *C*-terminal coiled-coil domains that play an important role in channel multimerization [15] as well as the interaction of TRPC channels with the endoplasmic reticulum (ER) Ca^2+^ sensor STIM1 [16]. TRPC members contains a *C*-terminal calmodulin (CaM)- and inositol 1,4,5-trisphosphate receptor (IP_3_R)-binding (CIRB) site, which participates in the regulation of TRPC channel function [17,18].

TRP channels are mostly non-selective cation channels that are permeable to both monovalent and divalent cations with Ca^2+^ to Na^+^ permeability ratios ranging from 0.01 to over 100 [19]. The pore-forming TM5–TM6 loop has been reported to be highly conserved among all TRP members, and contains several hydrophobic residues at the beginning of the channel pore. TRP channel gating occurs in response to a variety of physical and chemical stimuli and leads to both rises in cytosolic Ca^2+^ concentration and membrane depolarization, which, in turn, activate a number of cellular functions. TRP-induced membrane depolarization might also decrease the driving force for Ca^2+^ influx through other channels (see Section 3).

Since the identification of the mammalian TRP channels, a considerable attention has been focused on the role of TRPC1 and other TRPC channels as candidates to conduct Ca^2+^ influx during SOCE.

## 2. TRPC Channels in the STIM1–Orai1 Scenario

A new scenario emerged in the study of SOCE after the identification of Orai1 and Stim1 as the key components of the CRAC (Ca^2+^ release-activated Ca^2+^ channels). STIM1 was identified as the Ca^2+^ sensor in the ER which communicates the Ca^2+^ content of the stores to the channels in the plasma membrane, while Orai1 was identified as the pore subunit of the CRAC channel in the plasma membrane [38,39,40,41]. The expression of splice variants of STIM1 and Orai1 with functional and biophysical differences have been demonstrated in mammalian cells. STIM1L, a longer splice variant of STIM1 described in adult human muscle fibers, displays a fast full SOCE activation compared to STIM1 [42]. Regarding to Orai1, two different variants generated by alternative translation initiation, Orai1α and Orai1β, have been shown to drive *I*_CRAC_ and *I*_SOC_ currents [43,44]. In addition to these variants, mammalian cells also express other STIM and Orai isoforms involved in the generation of *I*_CRAC_ currents. STIM2 is a more sensitive ER Ca^2+^ sensor than STIM1, but it promotes a weaker CRAC channel activation [45]. Three variants of STIM2, (STIM 2.1, STIM2.2, and STIM2.3) with different roles in the modulation of SOCE have been identified. While STIM2.1 has been described to play an inhibitory role, STIM2.2 has been shown as an activator of SOCE. The function of the STIM2.3 variant still remains unclear [46,47]. Orai2 and Orai3 proteins have also been shown to drive *I*_CRAC_ currents after depletion of the intracellular stores [48,49,50] and their regulation and physiological role are less known as compared to Orai1. Therefore, it is currently widely established that the Orai-STIM complex, mainly Orai1-STIM1, constitutes the highly selective CRAC channel.

TRPC1 was the first candidate proposed as SOC channel in Chinese hamster ovary cells [51] and monkey COS cells [52] by the expression of TRPC1A, a splice variant of TRPC1, and the expression of a full-length cDNA encoding human TRPC1, respectively. In both cases, the consequence was an increased SOCE after depletion of the intracellular Ca^2+^ stores. Later, the role of TRPC1 as the SOC channel was confirmed using different approaches in a large number of human cells, including submandibular gland cells [53], endothelial cells [54] and platelets [55], among others. However, the involvement of TRPC channels in SOCE has long been controversial with different studies providing evidence against a functional role of TRPC channels in SOCE. For instance, overexpression of TRPC channels, including TRPC3 [56,57], has been found to induce non-capacitative Ca^2+^ entry downstream of phospholipase C in a variety of cell models. A major problem for the involvement of TRPC channels in SOCE is that these channels cannot reproduce the biophysical properties of *I*_CRAC_. Nevertheless, as *I*_CRAC_ is not the only store-operated Ca^2+^ current, this observation does not rule out the possibility that TRPC channels also participate in SOCE under certain scenarios, such as the assembly with the STIM1-Orai1 complex. In the new STIM1-Orai1 scenario for SOCE, it was soon reported that both proteins together with TRPC1 are assembled to form a dynamic STIM1-Orai1-TRPC1 ternary complex that drives the *I*_SOC_ current [22,58,59,60]. Upon store depletion, STIM1 activation promotes its oligomerization and translocation to the ER-PM junctions where it binds Orai1 [58,59] and TRPC1 [59,61,62] in lipid rafts domains, gating both Ca^2+^channels [63,64]. STIM1 mediates Orai1 activation by the interaction of the cytosolic STIM1-Orai1 activation region (SOAR) of STIM1 [24] with two STIM1-bindings sites located at the *C*- and *N*-termini of Orai1 [65,66,67]. The SOAR region is also required for STIM1-TRPC1 interaction; however, it is not sufficient to activate TRPC1 [24]. The activation of TRPC1 requires electrostatic interaction between highly positively charged lysines (^684^KK^685^) located in polybasic lysine-rich domain (K-domain) of the STIM1 *C*-terminus with the conserved, negatively charged, aspartate residues in TRPC1 (^639^DD^640^) and equivalent residues in other TRPC channels [25]. However, there is no evidence about the domains of Orai1 and TRPC1 involved in their interaction, suggesting that TRPC1-Orai1 binding could be indirectly mediated by STIM1 or still unidentified adaptor proteins [68,69]. 

The first evidence of the dynamic assembly of the STIM1-Orai1-TRPC1 ternary complex was found using immunofluorescence and confocal microscopy assay in human salivary gland cells. In resting conditions, STIM1 shows a diffused cytosolic localization while TRPC1 is located in the PM colocalizing with Orai1, although it is also expressed in the cytosolic region. After Ca^2+^ store depletion, STIM1 co-localized in the PM with both proteins, TRPC1 and Orai1, without modifying the TRPC1 and Orai1 colocalization [59]. Different studies have demonstrated that a functional Orai1 plays an essential role in the STIM1-Orai1-TRPC1 complex formation using different approaches. In human platelets, the STIM1-Orai1-TRPC1 ternary complex formation, including Orai1-STIM1 binding, was demonstrated using immunoprecipitation assays and the electrotransjection with an anti-Orai1 *C*-terminal antibody impairs the interaction between STIM1 and TRPC1, as well as SOCE activation after intracellular Ca^2+^ store depletion [58]. In Orai1 knockdown HEK-293 by siRNA-mediated gene silencing, cell transfection with the dominant negative mutants Orai1 E106Q or Orai1R91W, but not with a functional Orai1 construct, failed to restore SOCE [22,60]. Concerning Orai1 splicing variants, an elegant study demonstrated that both variants of Orai1, Orai1α and Orai1β, are equally involved in the generation of *I*_SOC_ currents in HEK-293 transfected with STIM1, TRPC1 and either Orai1α or Orai1β [43]. This finding suggests that the STIM1-Orai1-TRPC1 complex might include both Orai1α or Orai1β proteins.

**Figure 2:**
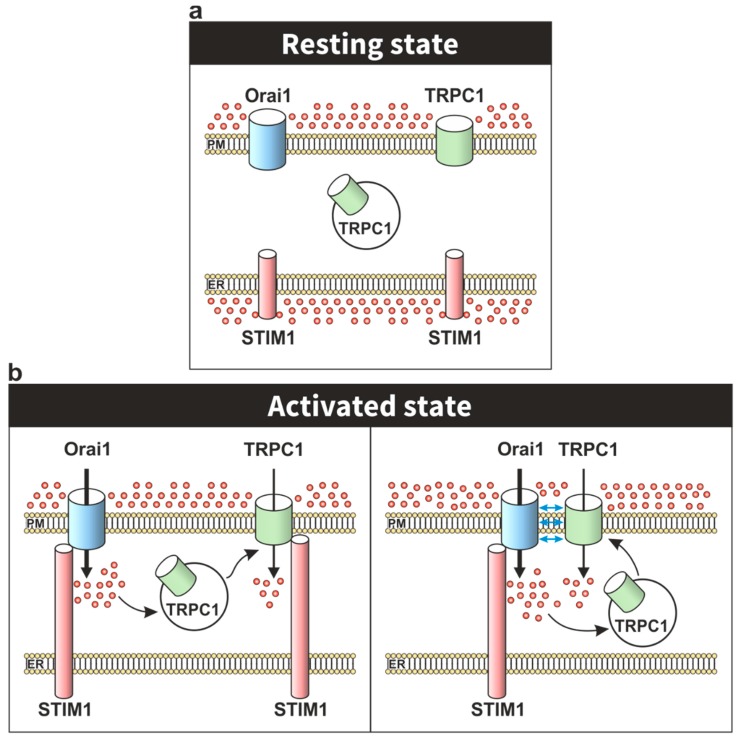
Cartoon depicting the activation of TRPC1 channels upon Ca^2+^ store depletion. (**a**) In the resting state, TRPC1 shows both plasma membrane and cytosolic localization. (**b**) Upon Ca^2+^ store depletion, Ca^2+^ influx via Orai1 has been reported to induce the translocation of intracellularly-located TRPC1 to the plasma membrane where it might be activated by STIM1. The model shows two alternatives for functional (mediating Ca^2+^ entry for the translocation of TRPC1 to the plasma membrane; left panel) or direct participation of Orai1 in the activation of TRPC1 (forming a STIM1–Orai1–TRPC1 ternary complex; right panel).

A model proposed by Cheng and coworkers, in human salivary gland cells, suggests that depletion of intracellular stores promotes Ca^2+^ influx via Orai1-STIM1 complex, providing a local increase in free Ca^2+^ concentration that induces the translocation of TRPC1 to the vicinity of the STIM1-Orai1 complex (Figure 2). Beyond the activation of TRPC1 by STIM1, this transition also leads to the association of TRPC1 and Orai1 in the same complex. Interestingly, this model could explain the essential role of Orai1 and the lack of strong evidence supporting the direct association between Orai1 and TRPC1 in the assembly of the STIM1-Orai1-TRPC1 complex [69]. Besides different biophysical properties, the Orai1-STIM1 complex to mediate the *I*_CRAC_ current and the STIM1-Orai1-TRPC1 ternary complex to mediate the *I*_SOC_ current also display specific temporal and spatial Ca^2+^ oscillatory patterns involved in the activation of different physiological functions and in the pathogenesis of a number of diseases (revised in [70]). For instance, Orai1-STIM1-mediated Ca^2+^ entry promotes the activation and nuclear translocation of the NFAT (nuclear factor of activated T-cells) transcription factor, while a TRPC1-dependent Ca^2+^ entry is responsible for NF-κB transcription factor activation in human submandibular gland cells [71]. STIM1-Orai1-TRPC1-mediated Ca^2+^ entry is also required for platelet aggregation [72], insulin release [73], adipocyte differentiation and adiponectin secretion [74], among other functions. Moreover, STIM1-Orai1-TRPC1-dependent Ca^2+^ currents have been associated to the Ca^2+^ mobilization responsible for the development of distinct cancer hallmarks in different cancer cell types, including prostate cancer cells [75] and colon cancer cells [76,77], while STIM1-Orai1-TRPC1-TRPC4-mediated Ca^2+^ currents are involved in the Ca^2+^ remodelling involved in hypertrophic cardiomyopathy in rat ventricular myocytes [78]. A more recent study has reported that in anterior pituitary (AP) cells from Orai1-lacking mice TG-induced SOCE as well as Ca^2+^ entry evoked by TRH and LHRH were impaired, by contrast, SOCE was unaffected in AP cells from mice lacking expression of all seven TRPC channels, although spontaneous intracellular Ca^2+^-oscillations associated to electrical activity as well as Ca^2+^ responses to TRH and GHRH were significantly reduced in the absence of TRPC channels, thus suggesting that SOCE might function independently of TRPC channels and that Orai1 and TRPC channels, such as TRPC1, might play different functional roles [79].

Despite the findings that proposed the STIM1-Orai1-TRPC1 ternary complex as the SOC channel, different observations suggest that ORAI1-STIM1 and TRPC1-STIM1 complexes can also drive *I*_SOC_ currents depending on the cell type and the components of its Ca^2+^ signalling toolkit. Hence in cells with a robust *I*_CRAC,_ such as Jurkat cells, the Orai1-STIM1 complex is involved in both *I*_CRAC_ and *I*_SOC_ currents [22]. Furthermore, different studies have shown that TRPC1 interacts with STIM1 forming a complex without the involvement of Orai1 to mediate SOCE in vascular smooth muscle cells with a contractile phenotype [80]. In human myotubes, where Orai1 has been reported to be essential for SOCE and differentiation [81,82], the TRPC1-TRPC4-STIM1L complex has been reported to form a SOC channel whose Ca^2+^ inward current is required for human myogenesis and to maintain fast repetitive Ca^2+^ release in human myotubes [83]. Interestingly, the integration of Orai1 in this complex promotes an enhanced *I*_CRAC_-like current involved in the development of the hypertrophic cardiomyopathy in rat ventricular myocytes, as described above [78].

## 3. Modulation of Orai1 Function by TRPC Channels

As mentioned previously, TRPC channels, especially TRPC1 [22,58,70,77] but also other members of the TRPC subfamily, such as TRPC4 [84,85] and TRPC6 [86,87,88,89], have been reported to conduct Ca^2+^ entry upon Ca^2+^ store depletion. However, there is a growing body of evidence indicating that TRPC channels play a more complex role shaping Ca^2+^ signals through Orai1 channels.

**Figure 3:**
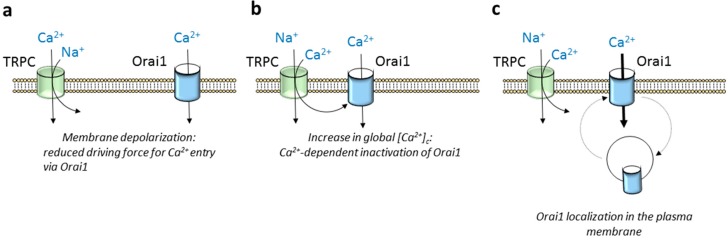
Overview of the modulation of Orai1 by TRPC channels. Orai1 channel function might be positively or negatively regulated by TRPC channels in the vicinity. (**a**) Ca^2+^ and Na^+^ entry through TRPC channels might lead to membrane depolarization and thus attenuation of the electrical gradient that favors Ca^2+^ influx via Orai1. (**b**) Ca^2+^ entry via TRPC channels participates in global rises in [Ca^2+^]_c_, thus leading to Ca^2+^-dependent inactivation of Orai1 channels. (**c**) Some TRPC channels are required for Orai1 recycling at the plasma membrane.

TRPC5 and TRPC6 show the greatest selectivity for Ca^2+^ relative to Na^+^ of the TRPC subfamily with Ca^2+^/Na^+^ permeability ratios around 9 and 5, respectively, while TRPC4 and TRPC1 are approximately equally permeable to Ca^2+^ and Na^+^ [90]. The latter means that TRPC channel gating leads to Ca^2+^ and Na^+^ influx in favor of an electrochemical gradient, which, in turn, might attenuate the inward flux of Ca^2+^ through Orai1 channels in two different manners: (1) inducing Ca^2+^-dependent inactivation of the Orai1 channels and (2) attenuating the driving force for Ca^2+^ entry as a result of membrane depolarization (Figure 3a,b). Concerning the first issue, fast Ca^2+^-dependent Orai1 inactivation has been suggested to be evoked by the interaction of Ca^2+^ entering through the channel itself to cytosolic inactivating sites in close proximity to the channel pore [91,92]; however, slow inactivation of Orai1 channels is associated to global increases in cytosolic Ca^2+^ concentration [93] that might be influenced by opening of TRPC channels in the vicinity of Orai1. In tumor cells with a gain of function of TRPC channels, in addition to Ca^2+^ entry, Na^+^ influx has been associated to Ca^2+^ efflux from the mitochondria due to exchange for Na^+^, thus resulting in further Ca^2+^-dependent inactivation of Orai1 channels (revised in [94]). Furthermore, the opening of TRPC channels might increase the amount of Ca^2+^ available to SERCA (sarco/endoplasmic reticulum Ca^2+^-ATPase) pumps and, therefore, store refilling, thus accelerating the deactivation of Orai1 channels. On the other hand, it has long been reported that TRP channel opening results in membrane depolarization. A well-known depolarizing TRP channel is TRPM4, which has been found to depolarize T lymphocytes [95]. Membrane depolarization induced by TRPC channel gating has been associated to a functional activation of voltage-dependent Ca^2+^ channels in electrically excitable cells [96,97]. In addition, depolarization evoked by Ca^2+^ and Na^+^ influx through TRPC channels leads to subsequent attenuation of the driving force for Ca^2+^ entry via Orai1 channels.

TRPC channels have also been reported to modulate the localization of other Ca^2+^-permeable channels in the plasma membrane. Schindl and coworkers have reported that co-expression of TRPC1 with TRPV6 down-regulates the plasma membrane expression of the latter [98]. TRPC channels has been found to be involved in the modulation of cytoskeletal rearrangements [99]. We have recently reported that TRPC6 modulates the plasma membrane expression of Orai1 and Orai3 channels in triple negative and luminal, respectively, breast cancer cells. Thus, attenuation of the expression of TRPC6, either by using interference RNA or by cell treatment with the phenolic compound oleocanthal, results in a significant decrease in SOCE in these cells [100,101]. TRPC6-dependent plasma membrane recycling of Orai1 is entirely dependent on Ca^2+^ and Na^+^ influx through TRPC6 channels as it is abolished by expression of the pore-dead dominant-negative TRPC6 mutant [100] (Figure 3c). Whether this mechanism is mediated by cytoskeletal remodeling remains to be determined.

## 4. Conclusions

TRP proteins form non-selective cation channels that play an important role in a variety of cellular functions and sensory transduction. The identification of STIM1 and Orai1 revealed the key components of the CRAC channels that mediate store-operated and highly Ca^2+^-selective currents. However, STIM1 and Orai1 alone are unable to support the store-mediated non-selective cation currents described in a number of cell types and that is when TRPC1 channels play an important role as constituents of the SOC channels. In addition to the role of TRPC1 in SOCE, TRPC channels also regulate the function of Orai1 in different manners, thus suggesting that TRPC channels play relevant functional roles in the STIM1-Orai1 scenario.

## Figures and Tables

**Table 1:** Biophysical features of store-operated Ca^2+^ channels. Notes: STIM1 CMD: STIM1 calcium modulating domain; DVF: divalent-free solution; n/d: not determined; STIM1 SOAR: STIM1Orai1-activating region.

	Orai1 Channels	Ora1-TRPC Channels	References
Current Voltage (I–V) profile	Inwardly rectifying	Inwardly rectifying	[20,21,22]
	Positive reversal potential ~ + 50 mV	Positive reversal potential 0 to ~ + 10 mV	
Permeability and Selectivity	Ca^2+^	K^+^, Na^+^, Cs^+^, Ca^2+^ and Ba^2+^	[4,23]
	Low to Cs^3+^		
	Conduct Na^+^, Li^+^ and K^+^ in DVF solutions		
Activation	Store depletion via STIM1 SOAR region	Store depletion via STIM1 SOAR and polibasic *C*-terminus regions	[24,25]
Endogenous current size	0.1–0.2 pA/pF at −100 mV		[26]
Fast Inactivation	Ca^2+^	n/d	[27,28]
	STIM1 CMD		
	Orai1 68–91 aa		
	Orai1 137–173 aa		
Slow inactivation	Mitochondria	n/d	[29,30,31]
	STIM1 390–391 aa		
	SARAF		
Inhibitors	2-APB (30–50 µM)	n/d	[32,33,34,35,36,37]
	La^3+^ and Gd^3+^ (100 µM)		
	Low pH = 6.7		
	Synta 66		
	GSK-7975A GSK-5503A		
	AnCOA4 (~5 µM)		
